# Maternal DNA lineages at the gate of Europe in the 10^th^ century AD

**DOI:** 10.1371/journal.pone.0193578

**Published:** 2018-03-14

**Authors:** Ioana Rusu, Alessandra Modi, Stefania Vai, Elena Pilli, Cristina Mircea, Claudia Radu, Claudia Urduzia, Zeno Karl Pinter, Vitalie Bodolică, Cătălin Dobrinescu, Montserrat Hervella, Octavian Popescu, Martina Lari, David Caramelli, Beatrice Kelemen

**Affiliations:** 1 Molecular Biology Center, Interdisciplinary Research Institute on Bio-Nano-Sciences, Babeș-Bolyai University, Cluj-Napoca, Romania; 2 Department of Molecular Biology and Biotechnology, Faculty of Biology and Geology, Babeș-Bolyai University, Cluj-Napoca, Romania; 3 Dipartimento di Biologia, Università di Firenze, Florence, Italy; 4 Faculty of History and Philosophy, Babeș-Bolyai University, Cluj-Napoca, Romania; 5 Brukenthal National Museum, Sibiu, Romania; 6 Department of History, Heritage and Protestant Theology, Lucian Blaga University of Sibiu, Sibiu, Romania; 7 Institute of Social Sciences and Humanities, Romanian Academy, Sibiu, Romania; 8 Department of Research-Development and Projects, Museum of National History and Archeology, Constanța, Romania; 9 Department of Genetics, Physical Anthropology and Animal Physiology, Faculty of Science and Technology, University of the Basque Country (UPV/EHU), Bizkaia, Spain; Ben-Gurion University of the Negev, ISRAEL

## Abstract

Given the paucity of archaeogenetic data available for medieval European populations in comparison to other historical periods, the genetic landscape of this age appears as a puzzle of dispersed, small, known pieces. In particular, Southeastern Europe has been scarcely investigated to date. In this paper, we report the study of mitochondrial DNA in 10^th^ century AD human samples from Capidava necropolis, located in Dobruja (Southeastern Romania, Southeastern Europe). This geographical region is particularly interesting because of the extensive population flux following diverse migration routes, and the complex interactions between distinct population groups during the medieval period. We successfully amplified and typed the mitochondrial control region of 10 individuals. For five of them, we also reconstructed the complete mitochondrial genomes using hybridization-based DNA capture combined with Next Generation Sequencing. We have portrayed the genetic structure of the Capidava medieval population, represented by 10 individuals displaying 8 haplotypes (U5a1c2a, V1a, R0a2’3, H1, U3a, N9a9, H5e1a1, and H13a1a3). Remarkable for this site is the presence of both Central Asiatic (N9a) and common European mtDNA haplotypes, establishing Capidava as a point of convergence between East and West. The distribution of mtDNA lineages in the necropolis highlighted the existence of two groups of two individuals with close maternal relationships as they share the same haplotypes. We also sketch, using comparative statistical and population genetic analyses, the genetic relationships between the investigated dataset and other medieval and modern Eurasian populations.

## Introduction

Dobruja is a historical region of Southeastern Europe, situated between the lower Danube River and the Black Sea. Today, this area is shared by Romania and Bulgaria, but in the historical past the territory surrounding the lower section of the Danube basin was a continuous territory and under the periodic domination and influence of distinct powerful state entities (Byzantine Empire, Kievan Rus’ and Bulgarians) [1, 2], being an important migration node between Asia and several parts of Europe. Within this area, one can find tracks marking the passage of multiple populations (*e*.*g*. Greeks, Romans, Byzantines, Pechenegs, Tatars, etc.) [1]. A witness of regional changes is the Capidava fortress, from the beginning of the 2^nd^ century AD when the Romans settled and became aware of its strategic importance, until the 11^th^ century when the Pecheneg invasion ended the Byzantine inhabitation of this location [3]. Capidava acts, from this perspective, as a gate of access for migratory populations towards Western Europe and also as a trade center with an extremely complex history during the Middle Ages. Through the 10^th^ century AD when Capidava is alternatively dominated, in the context of continuous conflicts between the Byzantine Empire, the Kievan Rus’ and the proto-Bulgarians, by each of them. Questions regarding the impact and extent of these past political and associated demographic events, that left imprints on the local genetic structure and influenced the current European gene pool remain unanswered, even though, the first elements of evidence could be inferred from the archaeological context [4]. Additional clues on historical population movements can be provided by ‘bio-archives’ such as human skeletal remains. Despite recent advances in the study of nuclear genome from ancient samples, mitochondrial DNA (mtDNA) remains a suitable instrument for studying past population movements [5–8].

Currently, most information on the genetic landscape of Romanian populations is based on mtDNA diversity in present-day inhabitants. A few studies attempted to evaluate their genetic relationship with other Eurasians [9–11], whereas some focused on mtDNA variation in minority groups [12–14]. To shed light on possible past migratory routes, a recent study pointed that contemporary distribution pattern of mtDNA haplogroups in the historical provinces of Romania is mostly governed by genetic affinities towards the Balkans, whereas the Transylvanian population is more closely related to Central European groups [15]. The genetic makeup of modern populations is the reflection of complex interactions between local and external genetic pools with variable dimensions and distributions. Traces of past events might have been blurred by more recent events, but direct evidence can be provided by local ancient genetic information. Ancient DNA (aDNA) studies using archaeological material from the current Romanian territory are scarce and restricted to distinct past periods such as the Early Neolithic, the Late Bronze Age [16] or the Upper Paleolithic which is represented by an early anatomically modern human fossil discovered in Peştera cu Oase, Southwestern Romania [17]. Thus, the genetics of Romanian medieval period remains a blank spot on the European map.

The aim of this study was to determine the genetic architecture of maternal lineages in a medieval population from Capidava, Dobruja, Romania, and also to evaluate the relationships to other medieval and modern populations from Europe and Asia. Medieval mtDNA information can be knitted together with archaeological and historical data to provide a better understanding of the impact of local events on the genetic structure of larger subsequent populations which are the *de facto* result of an original genetic pool modeled by familial relationships and immigrants arriving on different migratory routes, in different historical periods.

## Materials and methods

### Archaeological background and sample information

In this study we processed eleven archaeological human remains sets, identified as: Cap-M1, Cap-M2, Cap-M3, Cap-M4, Cap-M5, Cap-M6, Cap-M8, Cap-M9, Cap-M11, Cap_M15, Cap_M17, retrieved from the Capidava necropolis (zone X *extra muros*), in the Dobruja region, Constanța county, Capidava village, during the 2010–2014 campaigns. Yearly systematic archaeological research is carried out at this site under the coordination of the National History and Archaeology Museum—Constanța and authorized by the Romanian Ministry of Culture and National Identity through the National Commission of Archaeology. Permit numbers for the 2010–2014 campaigns are: 33/2010, 105/2011, 52/2012, 69/2013 and 45/2014. Reports detailing all archaeological excavations are yearly published in the *Cronica Cercetărilor Arheologice series*, edited by the National Institute of Heritage, in Romanian (cronica.cimec.ro). All human remains excavated from the X *extra muros* zone in this time frame were transferred to a permanent collection deposited for research at the Molecular Biology Center, Interdisciplinary Research Institute on Bio-Nano Sciences, “Babeș-Bolyai” University in Cluj-Napoca and carry the above mentioned codes. The collection is open to researchers, upon request.

**Fig 1:**
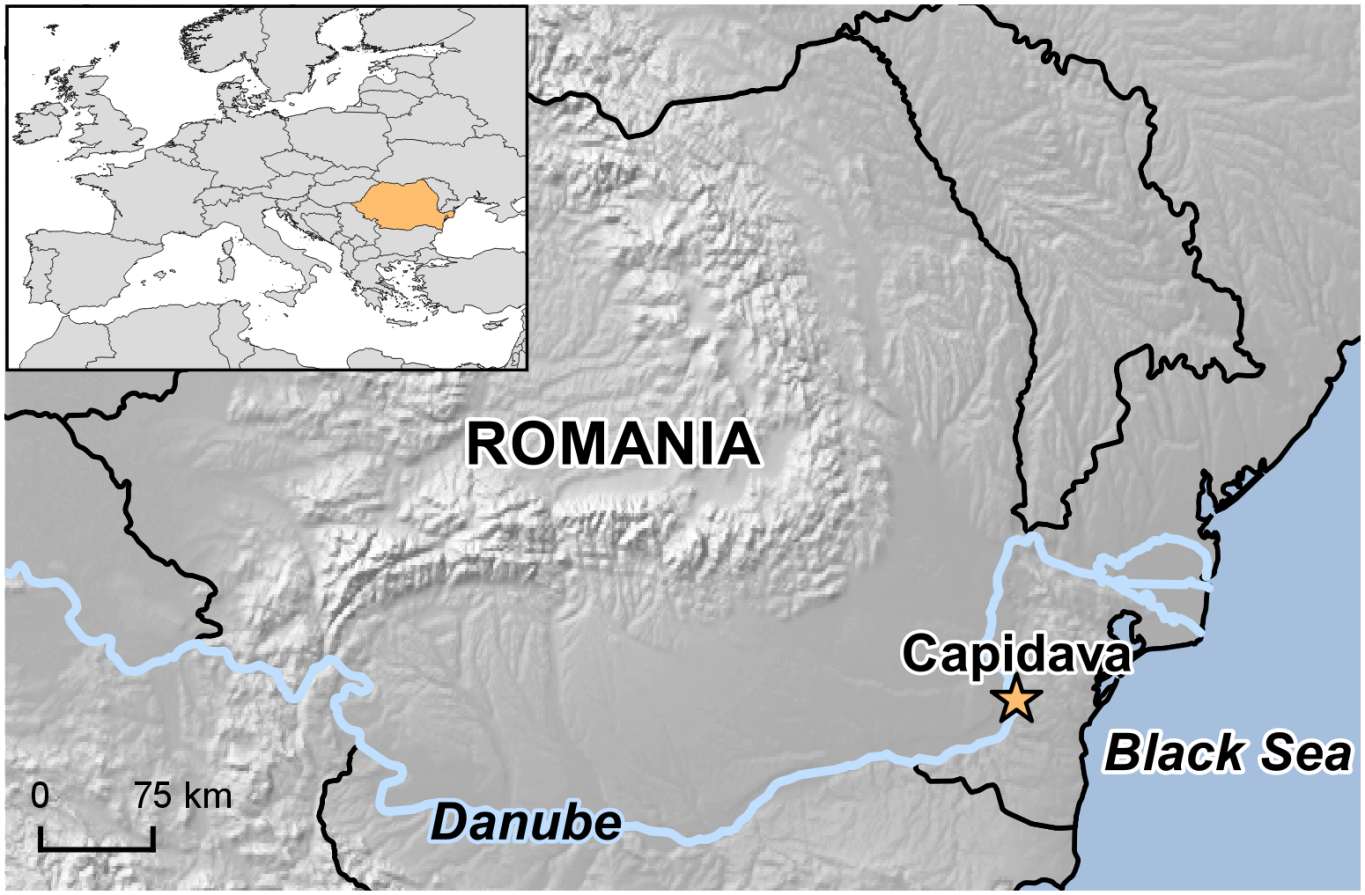
Location of the investigated medieval site. The map was created using QGIS 2.18.11.

We analyzed the human skeletal remains of 11 individuals excavated from the archaeological site of Capidava (Fig 1), outside the walls of the Capidava fortress, in its associated necropolis (S1 Fig). According to funerary rites, grave goods, and subsequent radiocarbon dating, the investigated individuals are dated to the 10^th^ century AD and were most probably part of a Christian population [4]. The only exception, the subadult from grave M6, placed in the same stratigraphic layer as the others, presented a particular and different funerary context marked by the evidence of a stone cist, being, therefore better fitted in the Roman period. A direct radiocarbon dating was performed on the skeletal remains of individual M4 at Beta Analytic (Florida, USA).

Prior to the molecular investigation, all specimens were morphologically assessed in order to infer sex, age at death, metric measurements and characterization of potential pathological conditions. For this purpose, standard guidelines were used [18, 19]. Of the total number of individuals (n = 15) recovered from the necropolis at the moment when this study was initiated, only 11 specimens were selected for the molecular analysis. This selection took into consideration the preservation status of the specimens. A summary of the data retrieved through the osteological and pathological analysis is listed in the S1 Table.

### Molecular analysis

#### Romanian laboratory

Molecular analysis of the 11 selected archaeological specimens was performed under sterile conditions in a dedicated ancient DNA facility at the Interdisciplinary Research Institute on Bio-Nano-Sciences, “Babeș-Bolyai” University, Romania, following strict guidelines and standard precautionary measures to ensure aDNA authenticity [20–23]. Laboratory rooms designated for pre-PCR and post-PCR processes are physically separated, and sterile conditions are maintained using UV-irradiation and regularly cleaning with bleach of all appliances, materials and work areas. The laboratory work was carried out while wearing suitable protective equipment that includes clean overalls, facemasks, shoe covers and disposable gloves. In order to screen for potential contamination with exogenous DNA, at least one extraction and one amplification blank for every three samples were used as negative controls. MtDNA profiles of the researchers who have been in contact with the samples were recorded and compared with the results obtained for the ancient specimens in order to track modern contamination (S10 Table).

Since teeth are less susceptible to contamination than other skeletal elements [24], intact ones (multi-rooted, if available) were selected as a source for aDNA isolation, for each tested individual. The external surface of the dental samples was decontaminated using bleach, washing with sterile water, followed by UV exposure (254 nm) in a cross-linker (UVILink CL 508G, UVITec, UK) for 25 minutes on each side. Biological material from human teeth was obtained by accessing the pulp cavity via the apical end of the roots using a dental micro-motor, at low speed (Marathon-3 Champion, Saeyang Microtech, Korea). The resulting powdered tissue was used for DNA extraction by means of a silica-based spin column protocol, modified after Yang et al. [25]. Generally, 100–200 mg of dental powder was incubated at 55°C for 40 hours with 1 ml digestion buffer (0.5 M EDTA pH 8.0; 0.5% SDS; 100 μg/mL Proteinase K), then incubated further at 37°C for 4 hours. This solution was afterward centrifuged at 2,000 x g for 5 minutes and the supernatant was divided into aliquots and transferred into new 2 ml Eppendorf tubes for DNA purification using QIAquick PCR purification kit (Qiagen, Hilden, Germany) according to manufacturer’s instructions. DNA was eluted in a final volume of 100 μl and subsequently stored at −20°C until use.

For results authentication, a second extraction was performed in the case of four samples (M1, M4, M8, M17), using an extraction method, designed in our laboratory, to co-purify DNA and proteins from archaeological remains. This protocol employs overnight decalcification of dental powder in 1.5 ml of 0.5 M EDTA solution pH 8.0 under permanent stirring, followed by the use of centrifugal filter units for concentration and purification of biological solutions (Amicon^®^ Ultra-15, MWCO 30,000 kDa, Millipore). The filtration product was separated into two equal aliquots, for specific downstream applications regarding DNA and proteins. Further purification of DNA was performed using the Animal and Fungi DNA Preparation Kit (Jena Bioscience, Jena, Germany) according to manufacturer’s specifications, resulting in a final total amount of 50 μl DNA extract.

Both hypervariable segments (HVS-I and HVS-II) of the mitochondrial genome were amplified using four overlapping fragments that span each of the HVS, with an average size of 141 bps [26]. Additionally, three primer pairs were used to amplify haplogroup (hg) diagnostic nucleotide positions (np) in the coding region (see S2 Table). PCR reactions were set up as follows: 2–4 μl DNA extract, 1.25 U MangoTaq^™^ DNA Polymerase (Bioline Reagents Ltd, London, UK), 1x MangoTaq^™^ Colored Reaction Buffer, 2.5 mM MgCl_2_, 0.2 mM dNTP mix, 0.5 μM each primer, and PCR-grade water (Jena Bioscience, Jena, Germany) up to 25 μl. The applied thermocycling conditions were: initial denaturation at 95°C for 5 min, 35 amplification cycles consisting of three steps (denaturation at 95°C for 30 sec, annealing at 46–56°C depending on the primer pair, for 30 sec and extension at 72°C for 30 sec) and final elongation at 72°C for 5 min. PCR products were checked on 1.5% agarose gel and then purified using FavorPrep GEL/ PCR Purification Kit (Favorgen Biotech Corp., Pingtung, Taiwan), according to the manufacturer’s protocol. All amplified target sequences were cloned (CloneJet PCR Cloning Kit, Thermo Scientific, Waltham, USA), and a minimum of six clones per amplicon was commercially sequenced (Macrogen Europe, Amsterdam, The Netherlands) using the standard primer pJET1.2R.

MtDNA sequences were aligned using BioEdit Sequence Alignment Editor v. 7.2.5.0 [27] in order to establish consensus sequence and to highlight *post-mortem* damage and/or potential contamination. HVS-I (np range 16009–16390) and HVS-II (np range 57–357) mutational motifs and coding region polymorphic positions were inferred by comparison to the revised Cambridge Reference Sequence (rCRS: NC_012920.1) [28]. Haplogroups were assigned using the HaploGrep2 web application, based on PhyloTree built 17 [29, 30].

#### Italian laboratory

For results authentication, a subset of samples (M2, M5, M9, M11, M15, M17) were independently processed in the Laboratory of Molecular Anthropology and Paleogenetics, University of Florence, Italy, a facility exclusively dedicated to ancient DNA investigations. Instead of using the classical method for the retrieval of genetic information from degraded samples, Next-Generation Sequencing (NGS) approach was used and the whole mitochondrial genome of the individuals was reconstructed.

DNA extraction was carried out using a commercial silica columns protocol designed to also recover very short DNA fragments, dominant in ancient samples [31]. Prior to DNA extraction, all dental pieces were cleaned by removing the outer surface with a micro-drill and UV-irradiation for 45 minutes in a cross-linker (UVILink CL 508G, UVITec, UK) and subsequently ground into a fine powder by a dental drill. Twenty microliters of each extract were then converted in a double-stranded and double-index Illumina library without enzymatic damage repair in order to preserve the damage patterns of the DNA fragments [32]. All libraries were amplified to reach the plateau and human mtDNA molecules were selected by capture with homemade probes [33]. Negative controls were set up during each experimental stage. High-throughput DNA sequencing was carried out at the Institute of Biomedical Technologies, National Research Council, in Milan. For this purpose, the Illumina MySeq platform was used and the sequencing was run as paired-end with 2 × 75 + 8 + 8 cycles.

A specific pipeline developed for the analysis of aDNA NGS data was used to retrieve the genetic information of the individuals from Capidava necropolis and to test for the authenticity of the obtained sequences. Primary data analysis was completed by means of the SeqPrep tool [34]: adapter sequences were trimmed and paired-end reads were merged into single sequences with a minimum overlap of 10 base pairs (bp), in order to exclude all sequences longer than 140 bp. Only reads with a minimum length of 30 bp were kept. Filtered reads were mapped against the rCRS using Burrows-Wheeler-Aligner (BWA) [35]; reads with mapping quality below 30 were discarded and PCR duplicates were removed by rmdup in SAMtools [36]. For all samples, a probabilistic iterative approach, Schmutzi [37], was used to call the endogenous consensus sequences and to estimate the contamination levels. Misincorporation patterns were tracked and quantified by mapDamage 2.0 [38]. The latter one was also executed for the reconstruction of endogenous mitochondrial genomes. Classification of complete mtDNA sequences according to haplogroup nomenclature was achieved using HaploFind [39].

#### Spanish laboratory

Molecular analyses for another subset of samples (M1, M3, M4, and M5) were replicated by another independent research group at the Department of Genetics, Physical Anthropology and Animal Physiology, University of the Basque Country UPV/EHU, Bizkaia, Spain. DNA was isolated from four teeth using the protocol based on guanidine thiocyanate and silica columns [40]. The extraction session involved two contamination controls that were applied to the entire process, except no tooth was added. The extraction and preparation of the PCR were undertaken in a specific lab for aDNA, equipped with a positive pressure sterile chamber, located in a physically separated space from the laboratory where post-PCR processes are carried out. All the work surfaces were cleaned regularly with sodium hypochlorite and irradiated with UV light. Suitable disposable clothing was worn (lab coat, mask, gloves, and cap).

The processing of the ancient samples in the UPV/EHU laboratory involved the application of a series of strict criteria for the authentication of results, as detailed by several authors [21, 41]. Sequencing of HVS-I (np 15998–16400) was undertaken in six overlapping fragments, each with a length of approximately 100 bp [16]. The amplification of each fragment was undertaken in independent PCRs. In the case of positive amplification and the absence of contamination, the PCR products were purified by ExoSAP-IT (USB Corporation, Cleveland, USA), with subsequent sequencing in an ABI310 automatic sequencer using chemistry based on BigDye 1.1 (Life Technology). The results obtained were edited with BioEdit software (http://www.mbio.ncsu.edu/BioEdit/bioedit.html) and the sequences were aligned manually [16].

Sequences corresponding to each medieval individual from Capidava were submitted to NCBI GenBank under the accession numbers MF320129-MF320142 and MF597774-MF597782.

### Reference population data sets

MtDNA profiles of medieval individuals from Capidava were compared with a data set of 15368 contemporary mtDNA sequences of the HVS-I region comprising European, Near Eastern and Asian populations. Another data set consisting of 495 medieval sequences corresponding to 13 populations originating from Europe and one from Asia was compiled in order to evaluate genetic relationships between the population analyzed in this study and other medieval populations. All sequence data were assembled from available published data, as listed in the electronic supplementary material S5 and S8 Tables.

### Population genetic analysis

Basic statistical methods were implemented for comparison and calculations of genetic distances between the examined population and other 14 ancient and 35 present-day populations. Diversity indices for the Romanian medieval population (consisting of 10 successfully typed individuals) were calculated in DNASP v5 [42] using both HVS-I (np 16009–16390) and HVS-II (np 37–357) sequences. Only 8 of the 11 individuals reported in this study were included in the population genetics analysis; reasons for this decision are outlined in the observation column of S3 Table.

Principal Component Analysis (PCA) was performed based on mtDNA haplogroup frequencies of medieval and modern populations (S5 and S8 Tables). Due to discrepancies in the phylogenetic resolution of the reference population’s mtDNA (haplogroup assignment based on older Phylotree versions) the haplogroup status in the case of these populations was updated, making them comparable to the genetic data reported in this study. In the PCA of the 15 medieval populations, 26 mtDNA haplogroups were considered (A, B, C/F/G, D, H, HV, I, J, JT, K, M, N1, N9, R0, R1, T, U, U2, U3, U4, U5a, U5b, U8, V, W, and X), while in the PCA of the 35 contemporary Eurasian populations, 36 mtDNA haplogroups were considered (A, B, C, D, F, G, H, HV, HV0, I, J, K, L, M, N, N9, R, T, T1, T2, U, U1, U2, U3, U4, U5a, U5b, U6, U7, U8, V, W, X, Y, Z, and Other). All PCAs were computed using the prcomp function of the built-in R stats package, R version 3.3.2 [43], and plotted in a two-dimensional space, displaying the first two principal components (PCs). In the case of the PCA comparing the Capidava medieval population to the modern-day populations, another graph displaying the first and third principal components was generated.

All PCs derived from the medieval PCA were used for hierarchical clustering, using Ward’s agglomerative method [44] and Euclidean distance measurement. The significance of each cluster was given as an approximately unbiased (AU) p-value, as a percentage, that was calculated using pvclust function [45] with 10000 bootstrap replicates. The resulted arrangement of the clusters was illustrated as a dendrogram in R 3.3.2 [43].

Inter-population comparisons were performed using the Arlequin software, version 3.5.2.2 [46]. Pairwise genetic distances (F_ST_) among populations were computed based on control region haplotypes of uniform sequence length, ranging from nucleotide position 16050 to 16383. Cytosine insertions at position 16193 were excluded from statistical analyses and all comparisons. The best fitting evolutionary model and the estimate of the gamma parameter were inferred from jModeltest 2.1.10 [47, 48]. Pairwise F_ST_ values were computed for both medieval and modern data sets, assuming a Tamura & Nei substitution model [49], 10000 permutations and an allowed level of missing data of 0.05 (S6 and S9 Tables). The linearized Slatkin F_ST_ values and significant p-values of the ancient populations (S6 Table) were visualized on a levelplot, whereas the matrix of linearized Slatkin F_ST_ values of modern populations (S9 Table) was displayed on a multi-dimensional scaling (MDS) plot represented in a two-dimensional space using metaMDS function based on Euclidean distances implemented in the vegan library of R 3.3.2 [43, 50].

Shared haplotype analysis (SHA) was performed in order to detect and compare the mtDNA haplotypes shared between the 15 medieval populations, by counting the absolute and relative shared haplotypes (S7 Table).

## Results and discussion

MtDNA sequences obtained in all three laboratories during this study (HVS-I and HVS-II in Romanian lab: M1, M2, M3, M4, M5, M6, M8, M9, M11, M15 and M17; HVS-I Spanish lab: M1, M3, M4 and M5 and Italian lab: M2, M5, M9, M11, M15 and M17), originating from the medieval Capidava necropolis are detailed in S3 Table. Only one of the individuals, M5, has a haplotype that matches the mtDNA profile of a researcher implicated in the experimental procedure, belonging to the common European J haplogroup. The only observed difference between M5 and researcher involves the absence of mutation 228A for M5, explained by the lack of amplification of segment ranging from np 169 to np 273 in the ancient sample. Even though the common mutations between M5 and researcher might be a coincidence, the fact that successful results were not obtained in either of the two additional laboratories in which M5 was processed indicates that it is prudent not to consider this sequence in the following statistical analysis. HVS-I of M1 and M3 was successfully replicated by the Spanish research group. In case of 5 individuals (M2, M9, M11, M15, and M17) the complete mitochondrial genome was reconstructed using high-throughput sequencing. All resulted sequences reach standard quality requested to guaranty the reliability of the NGS data (S4 Table). Whole mtDNA sequence data shows that all control region SNPs were correctly identified with PCR based methods. The DNA of the 3 remaining individuals (M1, M4, and M8) was extracted following two distinct protocols from different dental samples, most of the HVS-I fragments being amplified at least twice per each individual. All sequences that were replicated, along with the typed coding region positions, negative control results and the presence of characteristic *post-mortem* DNA damage in all cloned mtDNA inserts suggest the assigned haplotypes are authentic.

The 10 sequences that were successfully retrieved for the Capidava skeletal remains, spanning positions 37–357 and 16009–16390 belong to 8 haplotypes (haplotype diversity of 0.956). The unstable length polymorphisms associated with poly-C stretches between sites 302–315 has been ignored. Identical haplotypes were identified in two groups of two individuals derived from neighboring burials. One group of adult individuals that might be maternally related (M3 and M4) belong to hg R0a2’3, even though a first attempt to characterize their mitochondrial genetic material yielded a faulty, at that stage unverified assignment [51]. The other group consists of two subadults that possess the same haplotype (M9 and M11) belonging to hg N9a (S1 Fig). Due to the low frequencies of these haplogroups among ancient and modern populations, and the particular archaeological context, it is very unlikely that identical haplotypes occurred together by chance, given that cross sample contamination was excluded. The high number of intra-cemetery maternal relations relative to the population size suggests that family connections were defining parameters of spatial burial distribution. One possible scenario is that mobile groups of people that reached Dobruja settled for at least a few generations in the vicinity of Capidava fortress, burying their deceased in allotted familial cemetery plots. In order to avoid overrepresentation of mtDNA lineages due to potential family relationships, duplicate sequences were removed from the subsequent statistical analysis. This resulted in a medieval population consisting of 8 sequences with distinct haplotypes. Observation of diverse mitochondrial haplogroups in small medieval populations is prone to stochastic events.

The direct radiocarbon dating performed on the M4 human bones placed the remains at 880 and 990 cal. AD (Beta Analytic, Florida, USA) which corresponds to the age estimated according to the archaeological record. The medieval Capidava population analyzed here has a predominantly Western Eurasian haplogroup composition (H, R0, U3, U5, and V), the Eastern Eurasian component being represented by hg N9a with a frequency of 12.5% (S5 Table). In contrast to the other medieval populations analyzed in this study, the N9a haplotype is distinguished solely in the gene pool of the Capidava population. Characteristic N9a HVS-I polymorphisms have been identified in three human archaeological remains from the Carpathian Basin (Hungary), initially identified as early Neolithic (then revised as Sarmatian for two of the three samples, and Hungarian Conquest period for the third one) [52, 53]. Identical HVS-I haplotype was also found in an archaeological specimen (female) from Krasnoyarsk region, Siberia, dated to the Iron Age (100–400 AD) [54]. The presence of the N9a maternal lineage in the gene pool of medieval representatives from the Southeastern region of the extra Carpathian Basin (Dobruja) supports the idea of a “westward genetic influence of nomads from eastern Central Asia” [55]. N9a frequencies in modern European populations are extremely low and it is mainly found in the Volga-Ural region Tatars (~1%), Czechs (0.6%) and Russians from Southwestern Russia (1.5%), all potential donor or acceptor spaces for our population [56–58]. In our dataset of modern populations (S8), the highest frequencies (0.61%-1.61%) of N9 can be identified in three populations (KAZ, KGZ, and UZB) from Central Asia. Lower frequencies (0.13%-0.28%) of this maternal lineage can be observed in Eastern Europe (CZE, UKR) and in Southern Europe (ESP). The most frequent hg detected in Capidava population is H. This is the dominant maternal lineage among modern-day Europeans, encompassing over 40% of the total mtDNA variation in most of Europe [59]. However, its frequency observed in the analyzed medieval population is lower than it is in most contemporary European populations and in some historical Romanian provinces, other than Dobruja and Moldavia [8, 15]. It is important to note that the small sample size only allowed for rough comparisons.

Besides confirming the polymorphism pattern uncovered in the control region, the complete mitogenomic data of five individuals allows for the refinement of haplogroup assignment that was initially described by the hypervariable regions mutational motifs. According to the high-resolution mitogenomic data, M2 sample belongs to V1a maternal lineage, a typical Western Eurasian V sub-clade, that is currently distributed, based on complete modern mitochondrial data, across all over Europe and European Russia. Information from whole mtDNA sequencing reveals that this medieval sample matches two other ancient samples, one Early Bronze Age specimen from Germany (MF498721) [60] and a Neolithic one from Hungary (EBVO5a) [61]. The haplotype of M2 contains all of the expected V1a defining variants as well as three personal transitions (G9139A, T11089C, and C16266T). To our knowledge, none of these three polymorphisms were observed in conjunction with any published V complete mitogenome. As such, it occupies a nodal position within V1a, similarly to the two ancient published mitogenomes, and present-day samples from Western Europe. NGS data ascertain the fact that M9 and M11 have identical haplotypes, and due to the presence of the T2287C transition, they can be further classified as N9a9. Both samples harbor two private mutations (G228T and A15799G) which can be identified along with diagnostic motifs in two modern mitogenomes: FJ147307 [62], belonging to an individual from the North-East Altai, the other being associated with an individual from Kyrgyzstan KX675302 [63]. The common H control region haplotypes of M15 and M17 can be also further narrowed using the information from complete mitochondrial sequences. Sample M15 contains all defining mutations for H13a1a3, a descendant sub-lineage of the widely spread Western Eurasian H13 haplogroup. The H13a1a3 sub-branch currently includes only three modern maternally inherited genomes, two from Russia (KC911290 [64] and JQ701842 [65]) and one from Poland (JQ703193 [65]). Of them, the KC911290 sample from Belgorod region in Russia resembles the maternal signature of M15, having a genetic profile without private mutations. The matrilineal variability observed in the complete sequence of M17 can be classified as H5e1a1 and shows the presence of private mutation A13731G. This rare polymorphism (only 21 GenBank records in MITOMAP [66]; 0.049% when only full-length mitochondrial sequences are considered) is not presently associated with any H5e1a1 published mitogenome. This very derivative sub-branch currently includes only three modern whole mtDNA genomes, two from the European part of Russia (JX128081 and JX128091 [67]) and one from Denmark (KF161669). The further classification into V1a, H13a1a3, and H5e1a1 for the three Capidava samples points out to links much more geographically restricted to Central and Eastern Europe. This shows that even though few inferences regarding the genetic relations of the analyzed samples with modern and ancient mitogenomes can be made using only the control region, precise and direct connections among closely related mitogenomes can be identified based on high-resolution mitochondrial DNA data. This fact is even more conspicuous when N9a9 samples are taken into consideration. The genetic connection to Central Asia, already shown by the control region data, is now even more straightforward as both M9 and M11 share their two private mutations (G228T and A15799G) with two modern individuals, one belonging to the Tubalar ethnic group (Altai Republic) and one belonging to the Kyrgyz ethnic group.

**Fig 2:**
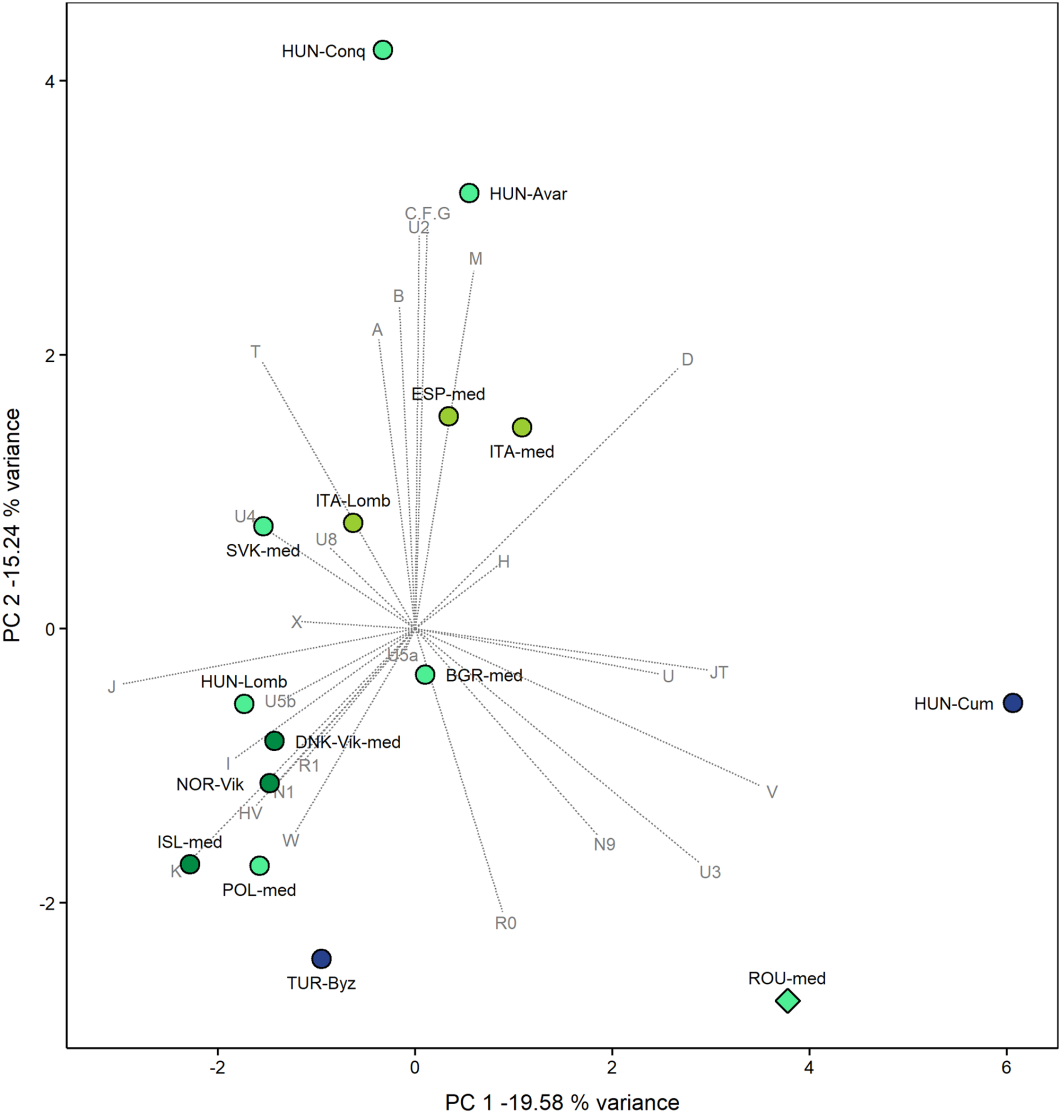
PCA plot of the first two components (34.82% of variance) of medieval populations. The PCA based on mtDNA haplogroup frequencies of the 15 medieval populations shows a roughly clustering of the ancient populations from Romania (ROU-med) and medieval Cumanians from Hungary (HUN-Cum). The other medieval populations from Southern Europe are clustered together, as are those from the North of Europe. The abbreviations, references and haplogroup frequencies are presented in S5 Table.

The PCAs of ancient and modern-day populations were computed based on mtDNA haplogroup frequencies (S5 and S8 Tables). The PCA of the 15 medieval populations considered here shows a roughly clustering between the investigated population and the Cumanians from Hungary along the PC1 and PC2 components (34.82% of the variance is displayed) (Fig 2). This assembly is fairly distantly positioned with respect to the remaining medieval European populations, being displayed at the opposite pole on PC2 from the Avars and the Hungarian conquerors. The farthest population along the PC1 component is the medieval population of Cumanians, culturally Asian steppe nomadic immigrants. Close genetic ties are shown between populations derived from same geographical areas, but this pattern is observed only for the medieval populations from Southern Europe and those from Northern Europe. Such connections were also previously noted by Csákyová et al [68]. Medieval populations from Eastern Europe are more dispersed within the PCA plot, the Romanian population being the most distant, fact which might be explained by a different hg composition, a potential sentinel signal for intense population movement in the area. For example, the genetic architecture of 13 proto-Bulgarians (BGR-med) shows no evidence of Central Asian maternal lineages, while the Western Eurasian maternal lineages do not match, with the exception of hg U3 and hg H, the mtDNA haplogroups found in Capidava [69]. A better understanding of the genetic links between past populations inhabiting modern Bulgarian and Romanian territories could be provided, in the future, by increasing the number of analyzed samples. Hierarchical clustering shows a very similar diagram to the PCA and reveals the clustering of the medieval Romanian population with the Cumanians from Hungary, a fact which can be explained by the interplay of all PCs in this analysis (S2).

**Fig 3:**
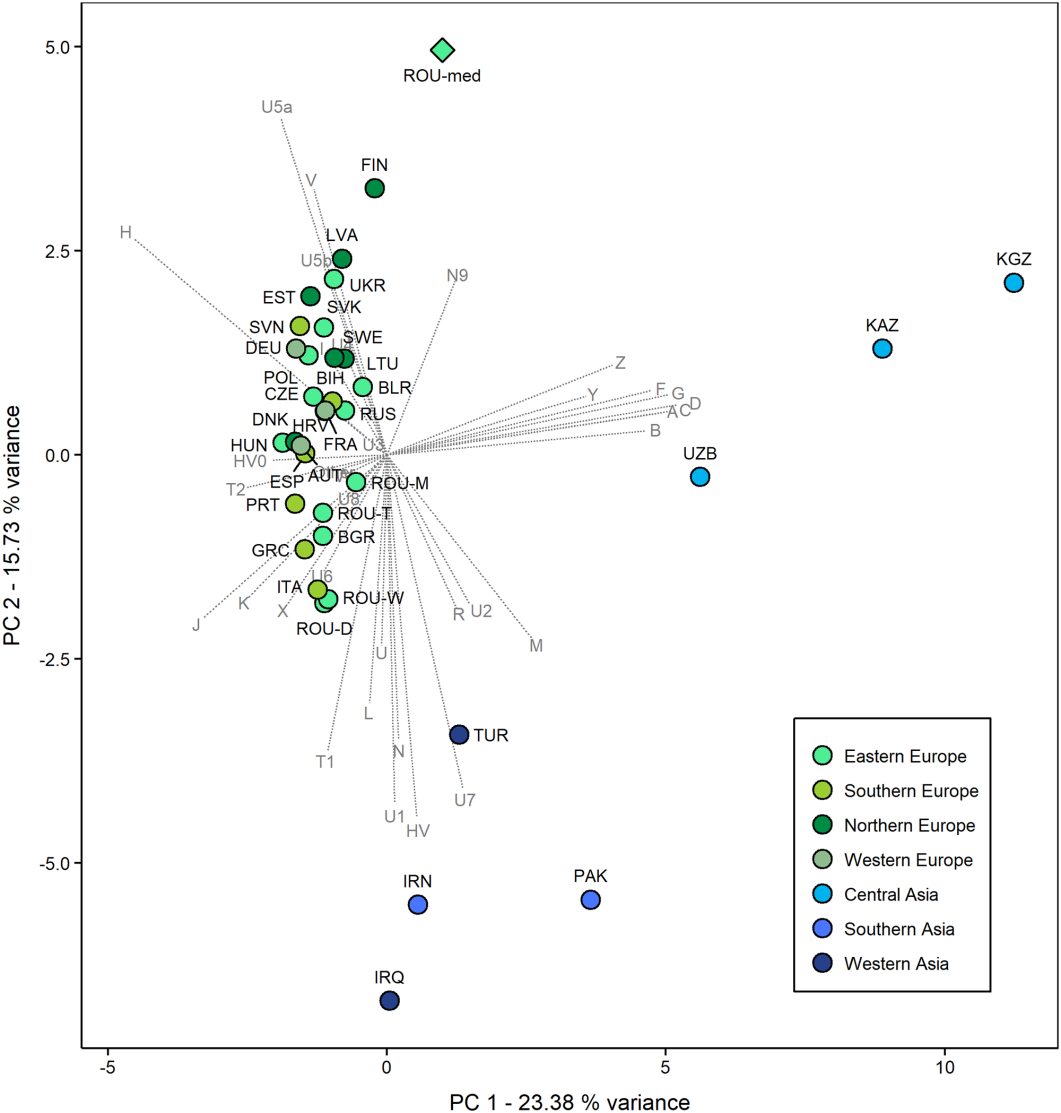
PCA of the investigated medieval and present-day populations. The PCA is based on mtDNA haplogroup frequencies of the medieval population from Romania and 35 modern populations from Eurasia and shows PC1 and PC2. The haplogroup frequencies and population information are listed in S8 Table.

The PCA plot of the contemporary Eurasian populations and the Capidava population shows the agglomeration of the European ones on the PC1 (23.38% variance) and PC2 (15.73% variance) components, in contrast to modern Asian populations which are scattered (Fig 3). Along PC1, the medieval Romanian population is more distant from the modern European groups than the Asiatic ones, but the contribution of the second PC shows that its connections to the latter populations are not so tight. Of the European populations, those that seem to have a higher genetic affinity with the one analyzed in this study, are mainly the Slavic populations from Eastern Europe, as well as those originating in the Northern Europe. Present-day Romanians inhabiting the historical province of Moldavia, an adjacent (to the North) region to Dobruja, displays closer ties to our medieval samples, indicating that this could have been a possible dispersal direction. Nevertheless, when PC3 is displayed (12.03% variance) the medieval Romanian population appears isolated from the rest of all modern-day populations due to dissimilarities induced by the haplogroups U5a, V, U3, N9 and R frequencies (S3).

**Fig 4:**
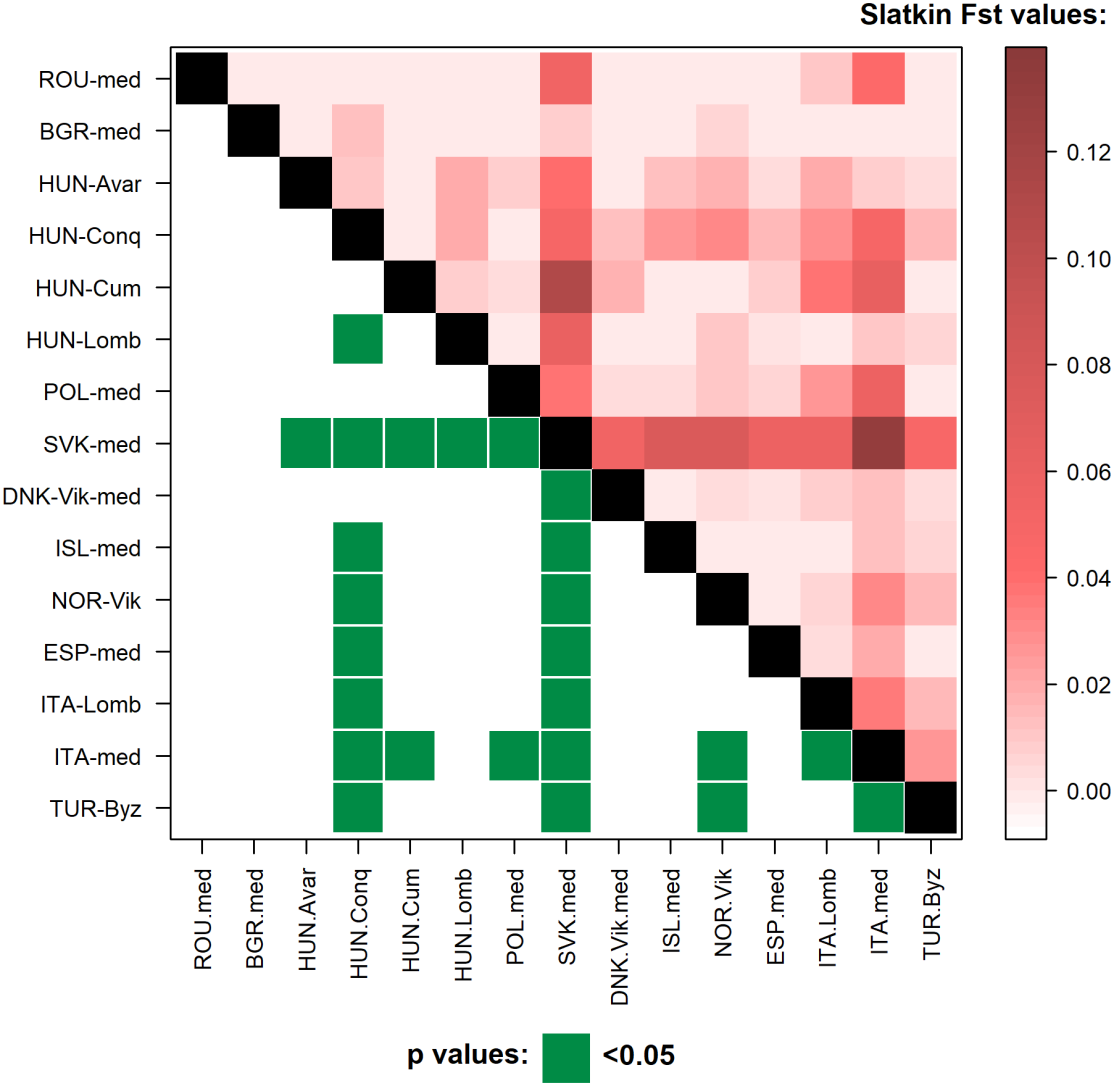
Levelplot of the linearized Slatkin population differentiation (F_ST_) values and significant p values. Lower left corner: significant p values (< 0.05) are indicated in green. Upper right corner: larger Slatkin F_ST_ values indicating greater genetic distances are marked by dark red shades. The exact F_ST_ and p values and population information are indicated in S6 Table.

Pairwise genetic distances were computed for 14 medieval populations *versus* the investigated medieval Capidava population and, respectively, a set of present-day populations from Eurasia. When compared to other populations dating from similar historical periods, the medieval population from the current Southeastern territory of Romania showed non-significant differences from all populations (p>0.05), the smallest p-values, associated to the highest F_ST_ values, being observed in medieval population from Slovakia (SVK-med) with p = 0.08336±0.0028 and F_ST_ = 0.05047 and in medieval Italians (ITA-med) with p = 0.12999±0.0036and F_ST_ = 0.04055 (S6 Table). The linearized Slatkin population differentiation (F_ST_) values and significant p values of ancient populations are visualized on the Fig 4 level plot.

**Fig 5:**
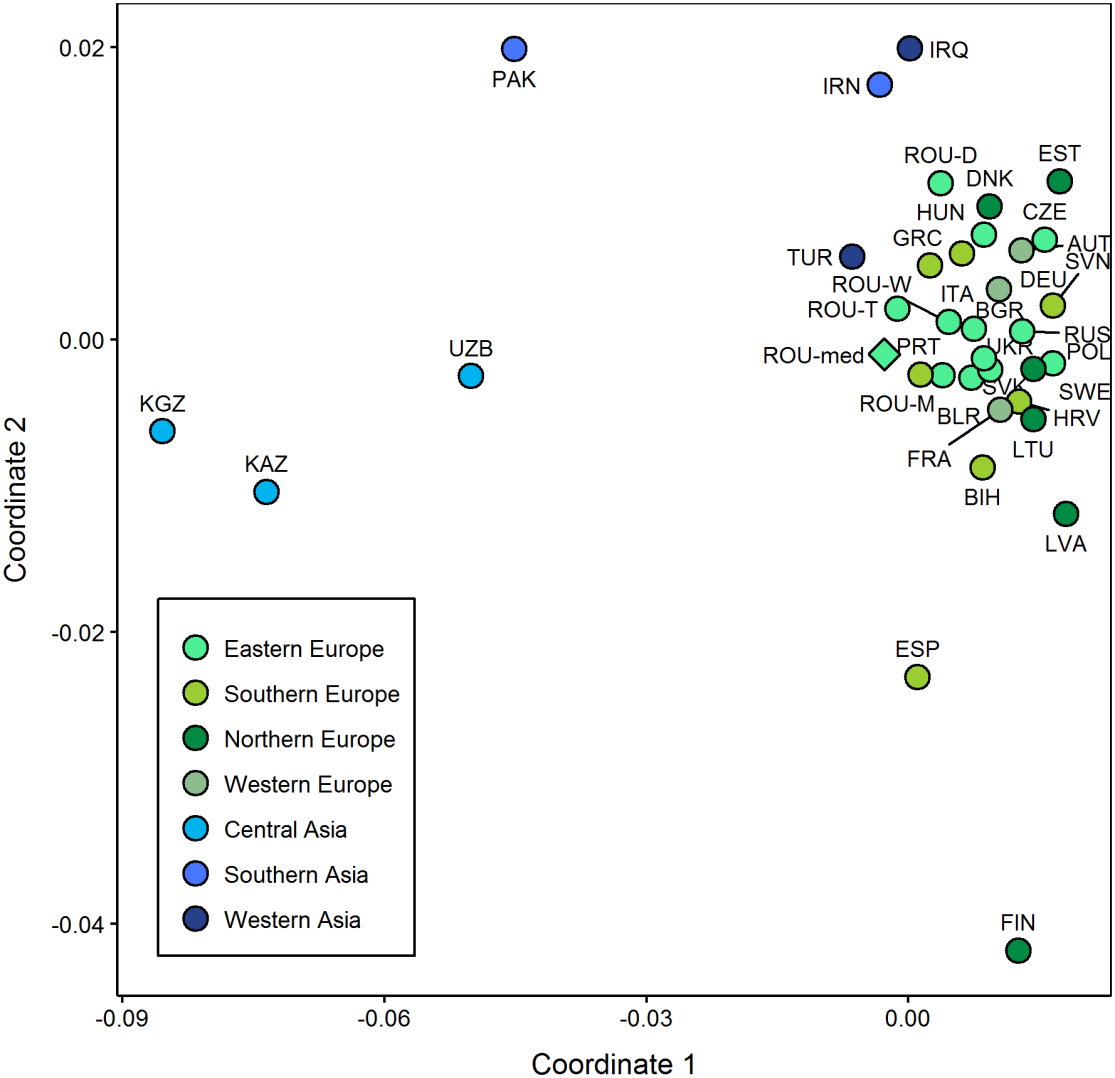
MDS plot of Romanian medieval population and modern Eurasian populations. Stress value is 0.1323 and non-metric fit (R^2^) is 0.982, values that highlight a good fit between the two-dimensional graph and the original distance matrix. The linearized Slatkin F_ST_ values and population information are presented in the S9 Table.

The medieval Romanian population showed the lowest genetic distances from present-day Slavic populations from Eastern Europe (BLR, UKR, RUS, SVN, and SVK) and also from a modern Turkish population (TUR), while the highest distances were noted relative to modern populations from Central and Southern Asia, as well as to current inhabitants of Dobruja. In either of these cases, the values were not significant (p>0.05) which is not quite surprising given the small sample size of the analyzed population and the different and very diverse haplotype composition. The exact F_ST_ values and their corresponding p-values are listed in the S9 Table. These genetic distances were visualized by displaying the linearized Slatkin F_ST_ values on a multi-dimensional scaling plot (Fig 5, S9 Table). This illustrates a similar pattern to the one reflected by the PCA, showing the aggregation of most of the modern European populations along the first two coordinates, whereas most of the Asian populations are disconnected. Of the Western Asian populations, the one from Turkey shows stronger affinities to the modern European ones and also to the Capidava population, a finding that can be somewhat explained by the prolonged interactions between the people from these regions (Turkic groups to which also the Cumanians and Pechenegs belong).

The SHA performed on the medieval populations reveals a distinct result from the F_ST_ calculation, showing that the medieval population from Romania shares most of the haplotypes with medieval populations from Spain and Italy (S4 Fig, S7 Table). This high proportion of lineage sharing between these populations, probably occurred due to the presence of the high percentage of the most widely distributed mtDNA haplotypes, H lineages, in modern populations of Eastern Eurasia, being therefore phylogeographically uninformative in this instance due to small data set and low HVS-I resolution. The strength of this apparent genetic affinity to medieval pools from Southern Europe is significantly diminished when high-resolution mitochondrial data is considered, as seen in the three Capidava samples (V1a, H13a1a3, and H5e1a1) that display strong, direct links to Central and Eastern Europe.

## Conclusion

The Capidava medieval sample analyzed here displays a very high haplogroup/haplotype diversity, which in conjunction with the very large difference when compared to the local modern population may indicate a very dynamic genetic environment: intense local population turnover. Genetic duplicates in adjacent burial plots highlight possible familial affiliations, characteristic for Christian cemeteries.

Low-resolution data obtained in this study suggest that in this particular case the haplogroups with low frequencies in modern populations (*e*.*g*. N9a) may be more informative than common genetic variants. The presence of the Central Asian N9a haplotype connected to ancient and conqueror Hungarian individuals, an ancient Siberian sample and modern Volga-Ural, Southwestern Russian, and Czech groups seem to place Capidava in a genetic landscape dominated by Turkic influences. Genetic affinities towards Central Asia are more straightforwardly illustrated by the high-resolution mitochondrial data as both M9 and M11 share their two private coding region variants with two modern individuals, one belonging to the Tubalar ethnic group (Altai Republic) and one belonging to the Kyrgyz ethnic group. The further classification of the common “European” lineages (H and V) into H5e1a1, H13a1a3, and V1a points out to genetic connections much more restricted to Central and Eastern Europe, decreasing thus the strength of the apparent link to medieval populations from Southern Europe which emerged from the analysis of low-resolution H haplotypes. Therefore, the phylogenetic and phylogeographic interpretation of the complete mitochondrial data, generated by NGS, counteracts to some extent the effects of the small sample size, emphasizing very precise and direct links among closely related old and modern mitogenomes.

Currently, different geographic regions and historical periods are characterized by imbalanced genetic datasets, be it due to number of samples/region or due to differences in resolution of genetic targets, thus limiting the establishment of strong unequivocal analogies. Given our small data set, the main importance of the present study consists in supplying a list of mitochondrial variants for a space and time completely lacking information, a situation similar to many other European medieval populations. In the age of NGS technology, a critical mass of data has to be reached in order to permit future more thorough phylogenetically, phylogeographically and demographically informative comparisons.

## Supporting information

S1 Fig Distribution of the graves within the Capidava necropolis. The colors of the graves symbolize the anthropological sex, age determination, and samples selected for molecular analysis.
(TIF)

S2 Fig Ward type hierarchical clustering of the medieval populations. Percentual AU p-values are given as red numbers on the dendogram.
(TIFF)

S3 Fig PCA plot of the investigated medieval and present-day populations. The PCA is based on mtDNA haplogroup frequencies of the medieval population from Romania and 35 modern-day populations from Eurasia, and shows PC1 and PC3. The haplogroup frequencies and population information are listed in the S8 Table.
(TIFF)

S4 Fig Levelplot of shared haplotype analysis (SHA). The levelplot is based on the percentage values of the relative shared haplotypes, also shown in the figure. The absolute values and the population information are given in S7 Table.
(TIFF)

S1 Table Information regarding the osteological and pathological analysis of the samples. (XLSX)

S2 Table Details of primer sets used for PCR. (XLSX)

S3 Table MtDNA results obtained in three different laboratories. (XLSX)

S4 Table Bioinformatics analyses output for NGS data. (XLSX)

S5 Table MtDNA haplogroup frequencies of medieval populations. (XLSX)

S6 Table F_ST_ values, p values and Slatkin F_ST_ matrix of the medieval populations. (XLSX)

S7 Table Shared haplotype analysis (SHA) of the medieval populations. (XLSX)

S8 Table MtDNA haplogroup frequencies of modern-day Eurasian populations. (XLSX)

S9 Table F_ST_ values, p values and Slatkin matrix of the analyzed and modern populations. (XLSX)

S10 Table Mitochondrial haplotypes of the researchers. HVS-I and HVS-II motifs of the researchers who had been in contact with the ancient samples.
(XLSX)
