# Supplementary figures and images for: Maternal DNA lineages at the gate of Europe in the 10th century AD
Source: PLoS One. 2018 Mar 14;13(3):e0193578. doi: 10.1371/journal.pone.0193578 (PMC5851556; doi:10.1371/journal.pone.0193578)

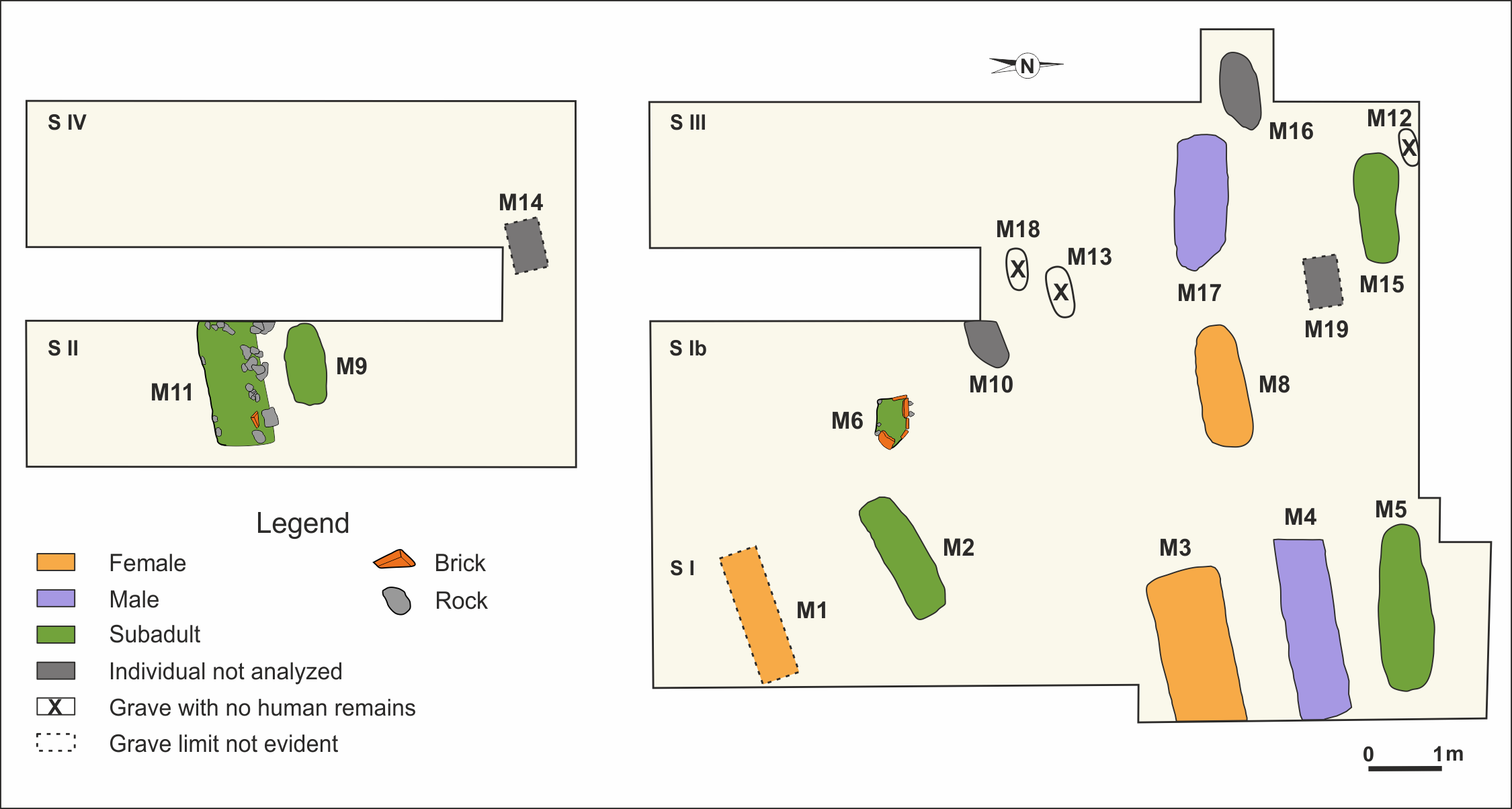

Supplement: S1 Fig — The colors of the graves symbolize the anthropological sex, age determination, and samples selected for molecular analysis. (TIF) [file pone.0193578.s001.tif]

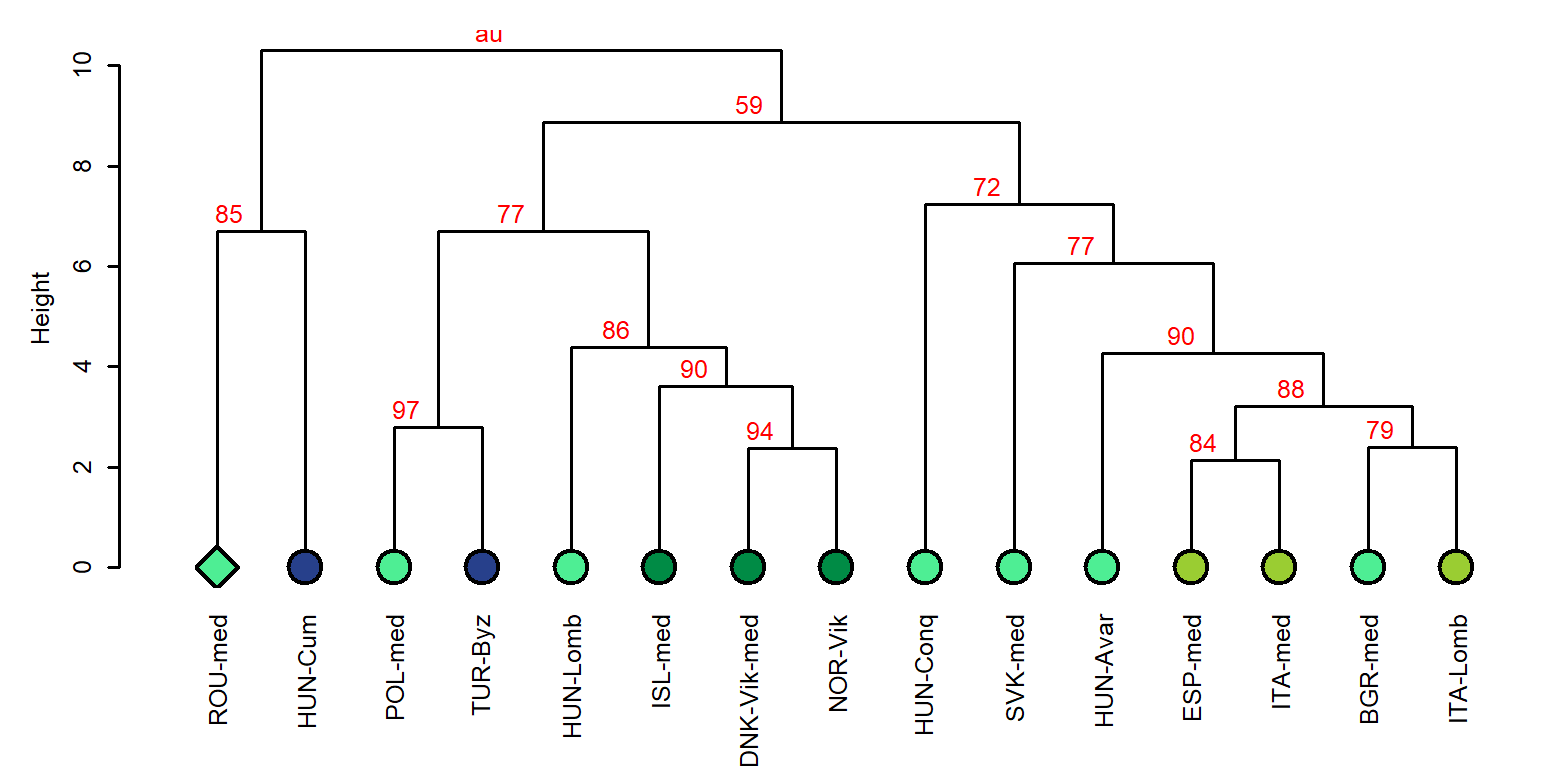

Supplement: S2 Fig — Percentual AU p-values are given as red numbers on the dendogram. (TIFF) [file pone.0193578.s002.tiff]

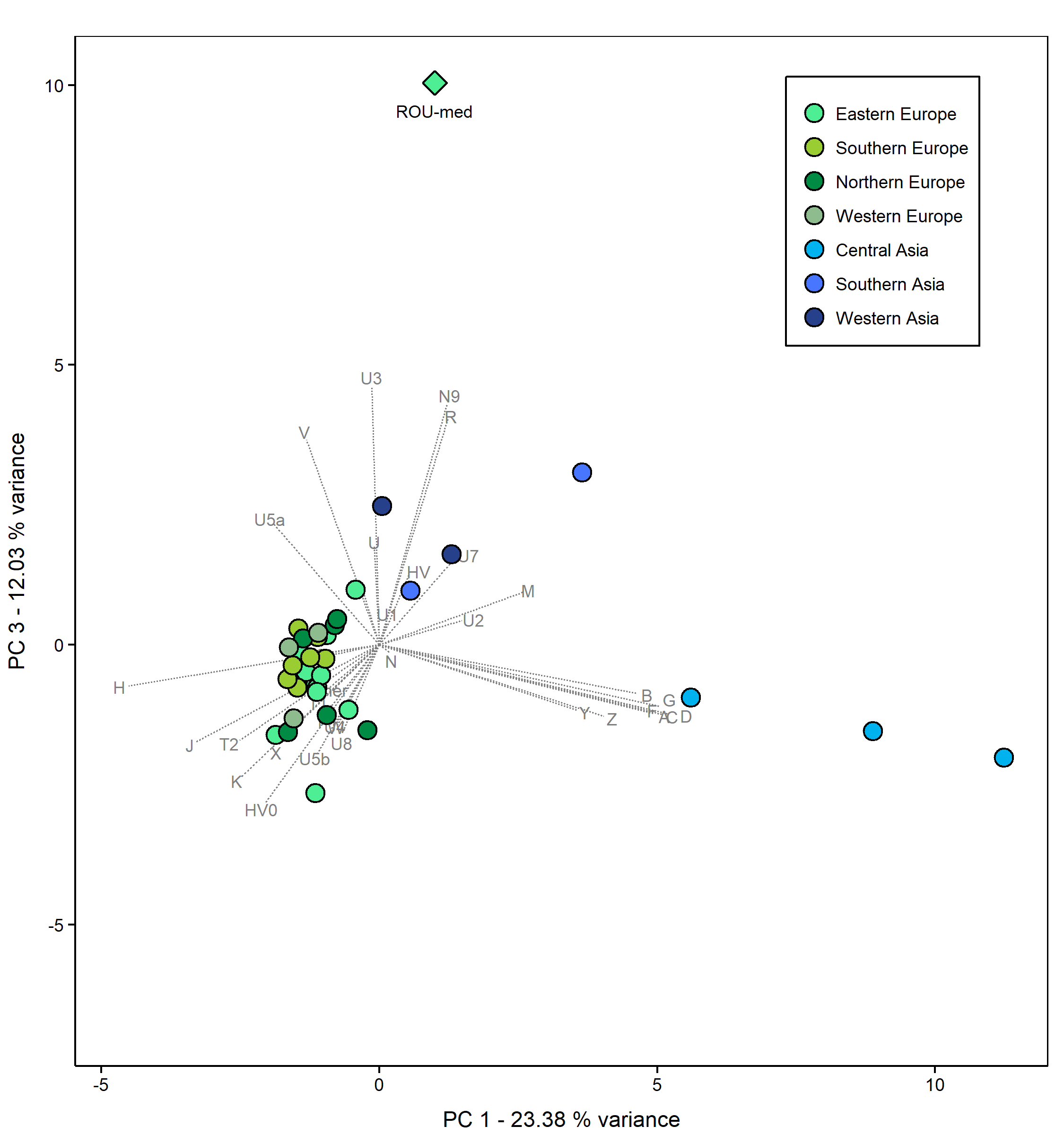

Supplement: S3 Fig — The PCA is based on mtDNA haplogroup frequencies of the medieval population from Romania and 35 modern-day populations from Eurasia, and shows PC1 and PC3. The haplogroup frequencies and population information are listed in the S8 Table. (TIFF) [file pone.0193578.s003.tiff]

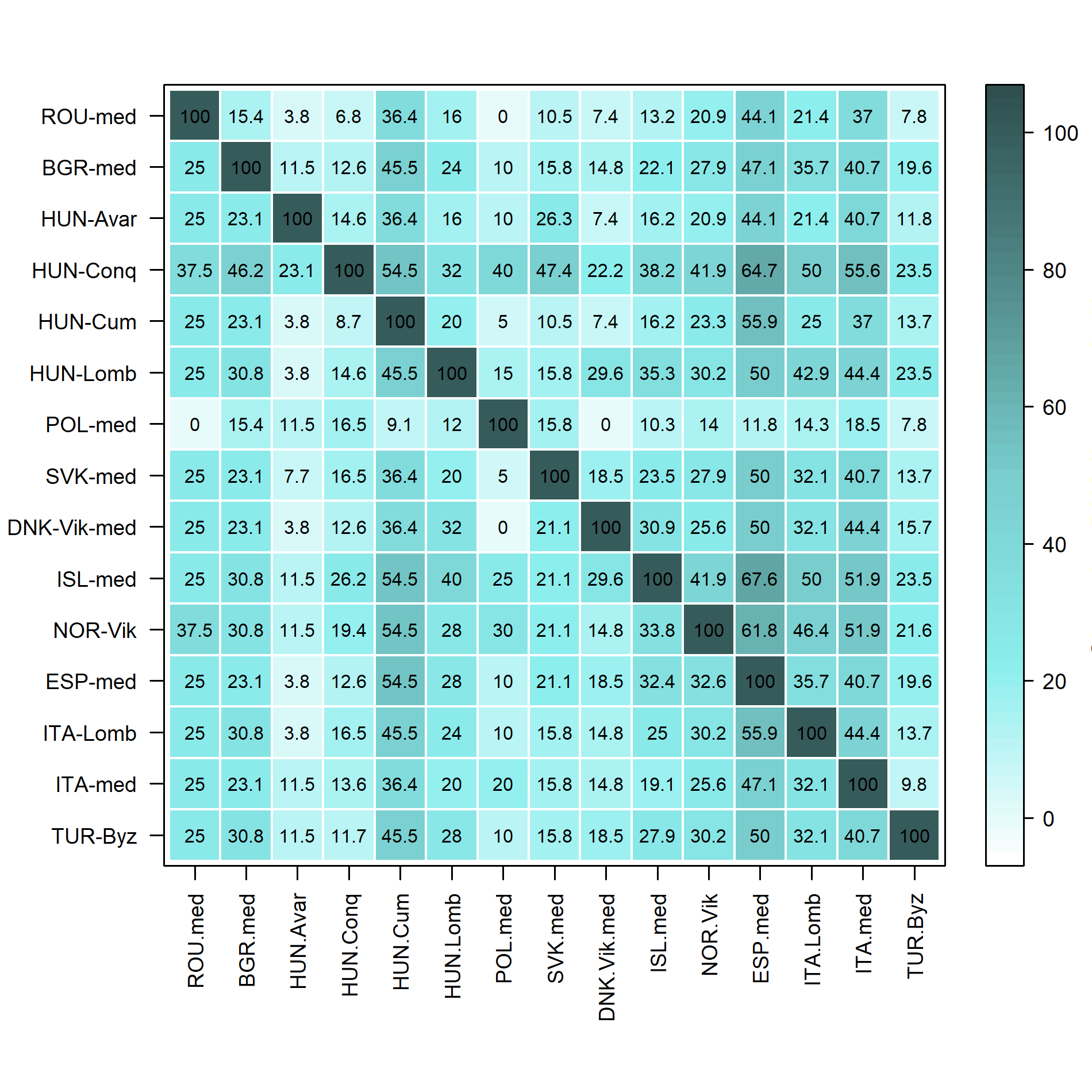

Supplement: S4 Fig — The levelplot is based on the percentage values of the relative shared haplotypes, also shown in the figure. The absolute values and the population information are given in S7 Table. (TIFF) [file pone.0193578.s004.tiff]
